# A Family with a Novel CTLA4 Haploinsufficiency Mutation and Neurological Symptoms

**DOI:** 10.1007/s10875-021-01027-1

**Published:** 2021-05-06

**Authors:** Alexandros Grammatikos, Sarah Johnston, Claire M. Rice, Mark Gompels

**Affiliations:** North Bristol NHS Trust, Bristol, UK

**Keywords:** CTLA4 haploinsufficiency, neurological symptoms, lymphoproliferation, autoimmunity, demyelination, immune tolerance

To the Editor,

CTLA4 haploinsufficiency is a rare autosomal dominant immune dysregulation disorder first described in 2014 [1, 2]. Patients with this disorder exhibit reduced expression of CTLA4, an inhibitory receptor that is found on activated and regulatory T lymphocytes, with subsequent T cell hyperactivation and lymphoproliferation. Here we report three members of the same family with a novel CTLA4 haploinsufficiency and neurological complications.

## Case 1

The index case presented with severe headaches at the age of 45. MRI brain revealed a focus of hypointensity in the frontal horn of the right lateral ventricle, which was stable on interval imaging. Past medical history and additional investigations are summarized in Table 1.

**Table 1:**
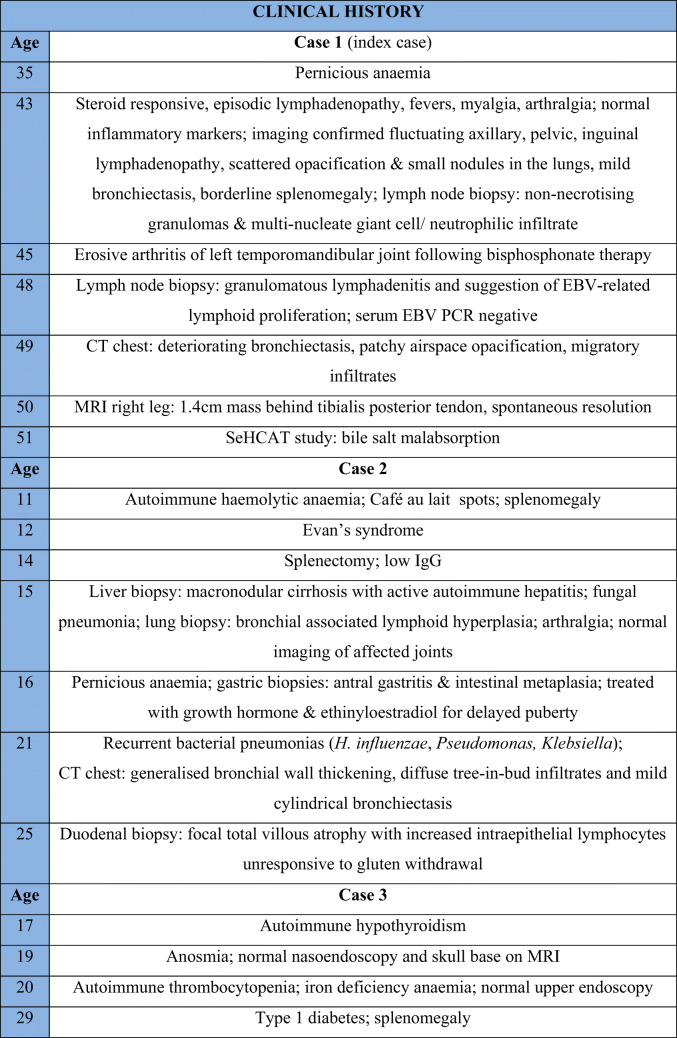

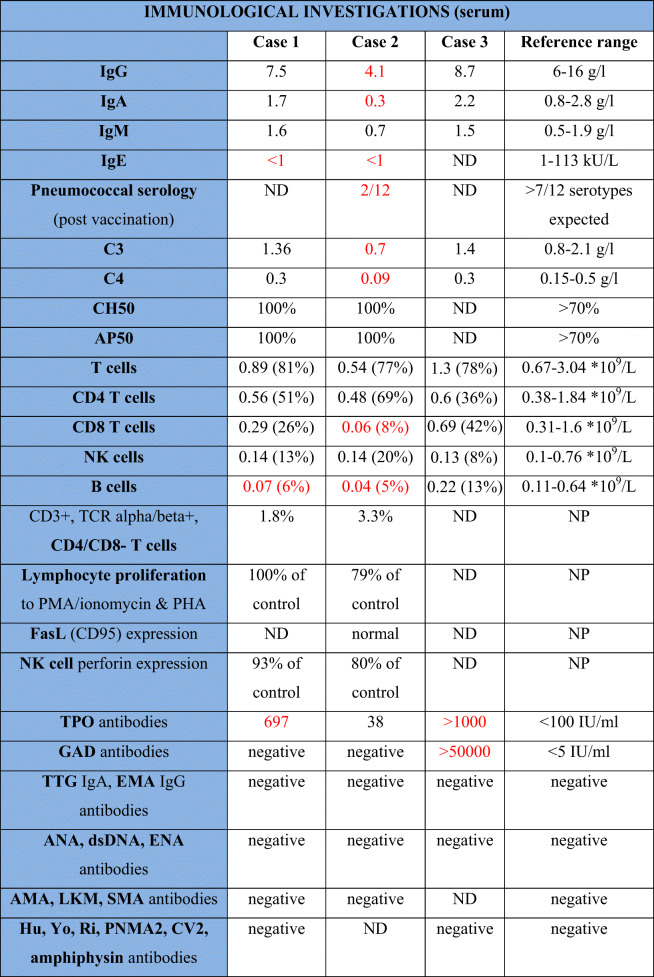

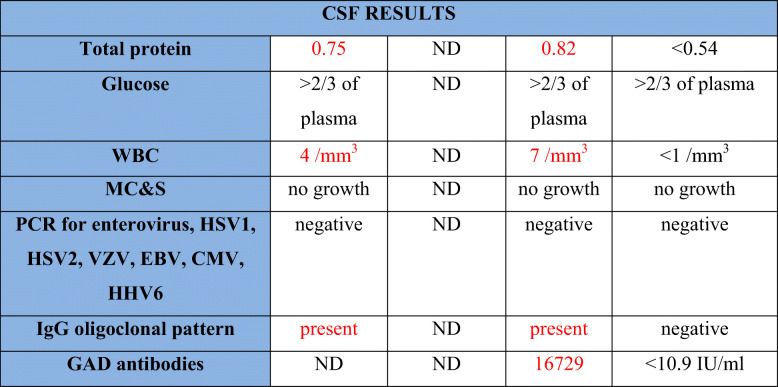
Clinical features and immunological investigations of presented cases

Family history (Suppl. Fig. 1) included two brothers with Evans syndrome (autoimmune thrombocytopenia and hemolytic anemia), a brother with unexplained lymphadenopathy, a brother who died at the age of 40 from left ventricular fibrosis, and a niece with recurrent cutaneous ulceration who was carrier for an LRBA mutation. Her eldest daughter developed acute myeloid leukemia at age 14, and her other two children are discussed below (Cases 2 and 3).

**Fig. 1:**
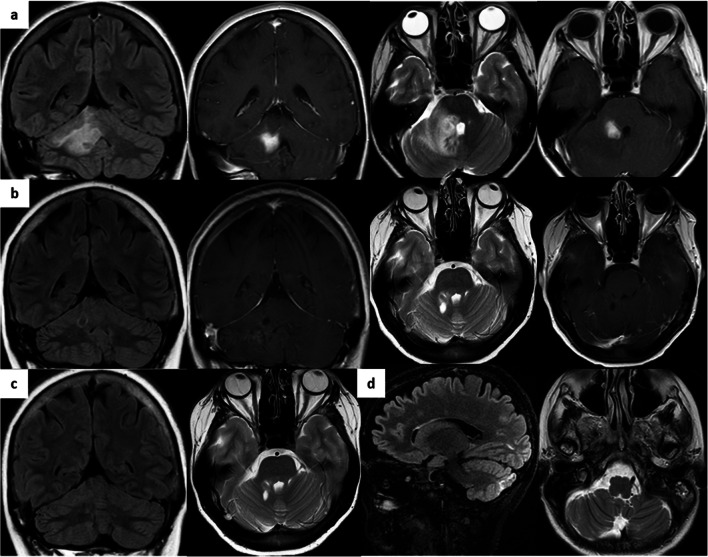
Brain MRI in cases 1 and 2. **a** Case 1 right middle cerebellar peduncular mass with surrounding edema (left, coronal views with and without gadolinium; right, axial views with and without gadolinium); **b** Case 1 reduction in the mass size post-treatment (left, coronal views with and without gadolinium; right, axial view with and without gadolinium). **c** Case 1 further reduction in the mass size leaving a small focus of presumed gliosis (left, coronal view; right, axial view); **d** Case 2 right inferior cerebellar high signal intensity (left, sagittal view; right, axial view)

At age 48, she developed left-sided numbness and weakness. Carotid ultrasound and echocardiogram were normal and the brain MRI scan was unchanged. A few months later, she presented with vertigo, vomiting, seizures, and right-facial dysesthesia, with dysarthria and right-sided dysmetria. Brain MRI now revealed a mass in the right middle cerebellar peduncle with surrounding edema (Fig. 1a). Cerebrospinal fluid (CSF) analysis demonstrated elevated protein and unmatched oligoclonal bands but no microorganisms. Symptoms responded to dexamethasone (16 mg daily) but relapsed on steroid wean (4 mg daily) when repeat MRI showed an enlarging mass. Histological analysis of the cerebellar peduncle mass was consistent with a florid active inflammatory and demyelinating process with neuronal sparing. The cellular infiltrate consisted of T cell lymphocytes, with a 2:1 ratio of CD4:CD8 T cells, plasma cells, and microglia [3]. Corticosteroids were restarted with clinical benefit, and a repeat MRI scan a few months later confirmed a radiological response (Fig. 1b).

Subsequent next generation sequencing of 194 genes associated with immune deficiency confirmed a heterozygous novel frameshift deletion in her CTLA4 gene, exon 1 (c.81dup encoding p.Leu28Serfs*32). This was confirmed by Sanger sequencing. In combination with the clinical picture and family history, a diagnosis of CTLA4 haploinsufficiency was made. She was started on sirolimus and the cerebellar peduncle lesion resolved leaving a small focus of presumed gliosis at the site of biopsy (Fig. 1c). No interval change has been seen on MRI over the following 3 years but the patient continues to experience focal seizures.

## Case 2

This patient was the second daughter of the index case. She had her first hospital admission at the age of 10 months, with suspected viral meningitis. Her subsequent clinical course is set out in Table 1. At the age of 12 years, she presented with severe headache, and brain MRI revealed a high signal intensity lesion in the right cerebellar hemisphere (Fig. 1d) and left superior frontal gyrus. She was treated with high-dose oral corticosteroids for presumed central nervous system (CNS) inflammation. At age 15 years, she presented with recurrent episodes of headaches, peripheral paresthesia, and muscle cramps and was again treated with corticosteroids.

She was referred to adult immunology at the age of 18 years, and investigations revealed two benign polymorphisms in her perforin gene. Genetics for autoimmune lymphoproliferative syndrome were however negative (Fas, Fas ligand, Caspase 10, Caspase 8, NRAS genes).

Following her mother’s diagnosis of CTLA4 haploinsufficiency, she was confirmed to have the same genetic mutation. She is now on immunoglobulin replacement for hypogammaglobulinemia with corticosteroid and azathioprine to manage autoimmune hepatitis.

## Case 3

This 32-year-old male, son of the index case, has type I diabetes and hypothyroidism. From the age of 29 years, he developed headaches, nausea, memory impairment, poor coordination, as well as olfactory and auditory hallucinations. MRI brain scans, including venography, were normal and there was no enhancement with gadolinium. CSF analysis revealed a mild lymphocytosis with raised CSF protein (Table 1). Unmatched oligoclonal bands were detected on one occasion but were not persistent. Electroencephalography confirmed complex partial seizures arising from the right hemisphere and occurring on a background of mild excess of nonspecific slow and theta activity. Anti-N-methyl-d-aspartate receptor and voltage-gated potassium channel antibodies were negative, but anti-thyroid peroxidase (TPO) and glutamic acid decarboxylase (GAD) antibodies were detected. Subacute memory impairment, altered personality, and psychiatric symptoms together with the emergence of seizures, CSF pleocytosis, and autoantibodies (anti-GAD) were in keeping with autoimmune encephalitis [4]. He was treated with corticosteroids, plasmapheresis, and anticonvulsant therapy with introduction of azathioprine and then mycophenolate as a steroid-sparing agent. Following the diagnosis of CTLA4 haploinsufficiency (age 32 years), he was switched to sirolimus with some improvement to his symptoms; headaches and hallucinations resolved, seizure control improved, and there has been no further decline in cognitive function.

## Discussion

Haploinsufficiency mutations in CTLA4 are known to be pathogenic. To date, 45 mutations have been described in the CTLA4 gene; 8 in exon 1, 31 in exon 2, and 6 in exon 3 (Suppl. Fig. 2). This family has a novel mutation, involving duplication of a nucleotide in exon 1 of the CTLA4 gene (cDNA position 81), with a predicted amino acid change of Leu28Serfs*32. This results in a cDNA frameshift and the introduction of a stop codon a short distance downstream, with significantly truncated CTLA4 protein.

A heterozygous variant of uncertain significance in LRBA exon 42 c.6424 T > C, encoding p. Phe2142Leu, was also detected in the index case and in her niece (patient with recurrent cutaneous ulceration; Supplementary Fig. 1). Heterozygous LRBA mutations are not known to be pathogenic and indeed LRBA protein expression was found to be normal in both patients. Another brother has recently presented with seizures and steroid-responsive CNS infiltration; genetic testing confirms the familial CTLA4 mutation (Supplementary Fig. 1).

These cases illustrate phenotypic variability associated with CTLA4 haploinsufficiency. Symptoms have been reported from infancy to adulthood, with a median age of 11 years [5]. Lymphoproliferation is the most common manifestation (73%), followed by autoimmune cytopenias (62%), and respiratory (68%) and gastrointestinal (59%) symptoms [5]. Neurological complications have been reported in 28% of patients [5], and histologically confirmed CNS inflammation has been previously described [6].

Although the role of CTLA4 in the CNS is unknown, reduced CTLA4 function is expected to lead to neuroinflammation. Interestingly, patients with multiple sclerosis (MS) have reduced CTLA4 expression [7], while certain CTLA4 polymorphisms are linked to reduced remyelination in MS [8]. Encephalitis and demyelination have also been reported in patients treated with ipilimumab [9, 10], a CTLA4-blocking monoclonal antibody used in the treatment of melanoma. Lymphoproliferation with demyelination and mass effect largely explain the observed neurological features in the index case. The etiopathogenesis in case 3 could be autoimmune; e.g., anti-GAD antibodies are known to be associated with limbic encephalitis, stiff person syndrome, and ataxia.

Overall, these cases highlight varied neurological sequelae associated with CTLA4 haploinsufficiency beyond lymphocytic infiltration, to include autoimmune-mediated damage within the CNS.

## Supplementary Information

ESM 1 (DOCX 82 kb)

## Data Availability

Not applicable.
