# Supplementary material for: A Family with a Novel CTLA4 Haploinsufficiency Mutation and Neurological Symptoms
Source: J Clin Immunol. 2021 May 6;41(6):1411–6. doi: 10.1007/s10875-021-01027-1 (PMC8310858; doi:10.1007/s10875-021-01027-1)
Supplement: Supplementary file 1 — (DOCX 82 kb) [file 10875_2021_1027_MOESM1_ESM.docx]

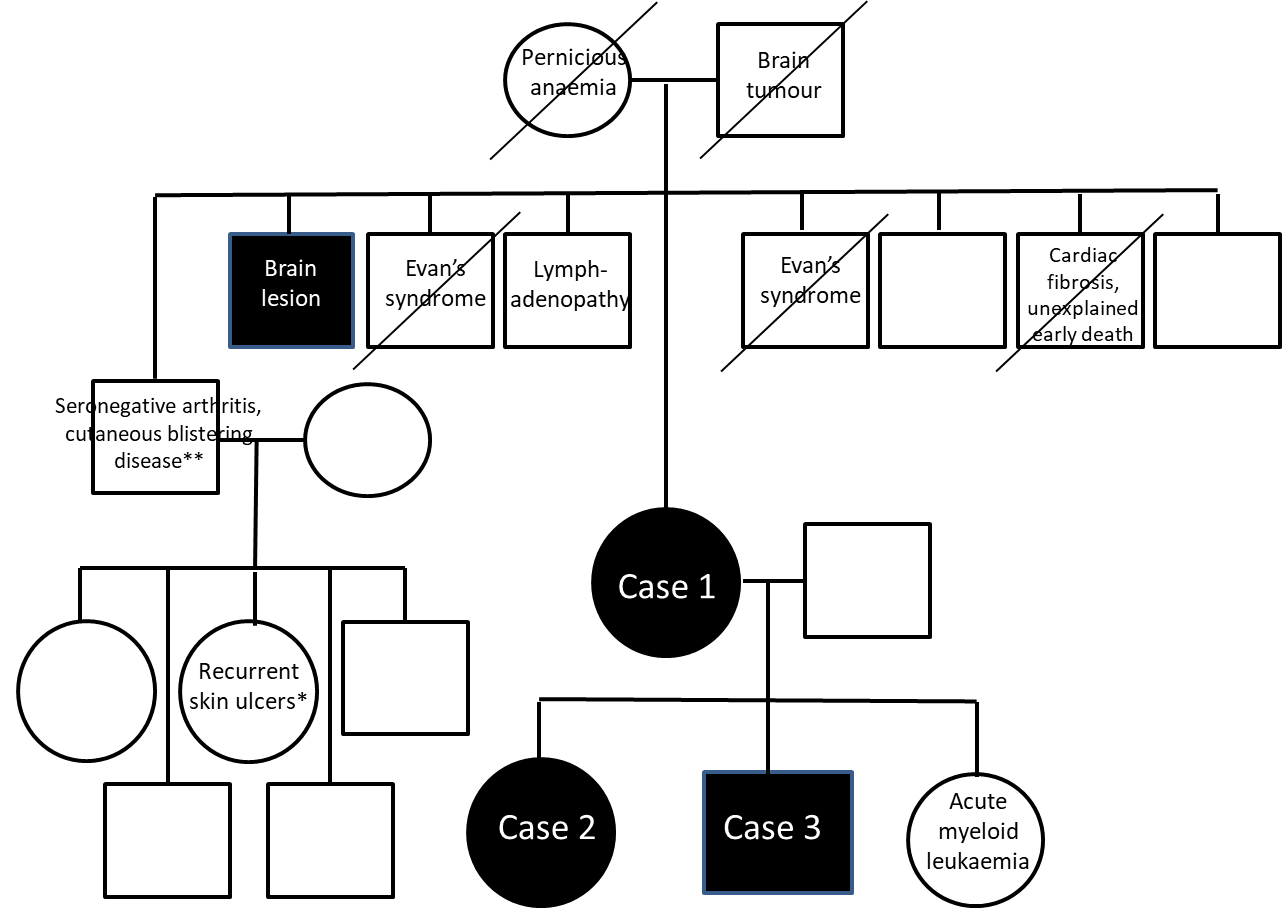


**Supplementary Figure 1.** Family tree of reported cases *= patient tested positive for a heterozygous LRBA mutation but negative for the CTLA4 mutation. **= patient tested negative for the CTLA4 mutation.


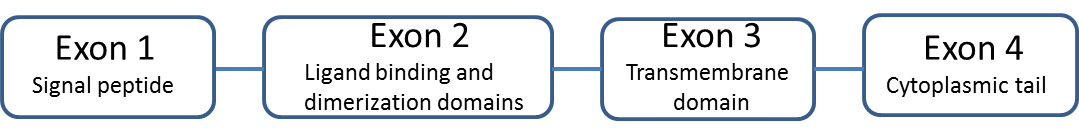


**Supplementary Figure 2.** CTLA4 gene structure**.**
